# Supplementary material for: Divergent Contribution of Cytoplasmic Actins to Nuclear Structure of Lung Cancer Cells
Source: Int J Mol Sci. 2024 Dec 19;25(24):13607. doi: 10.3390/ijms252413607 (PMC11727787; doi:10.3390/ijms252413607)
Supplement: Supplementary file 1 [file ijms-25-13607-s001.zip › ijms-3317929-supplementary.pdf]

Supplementary data

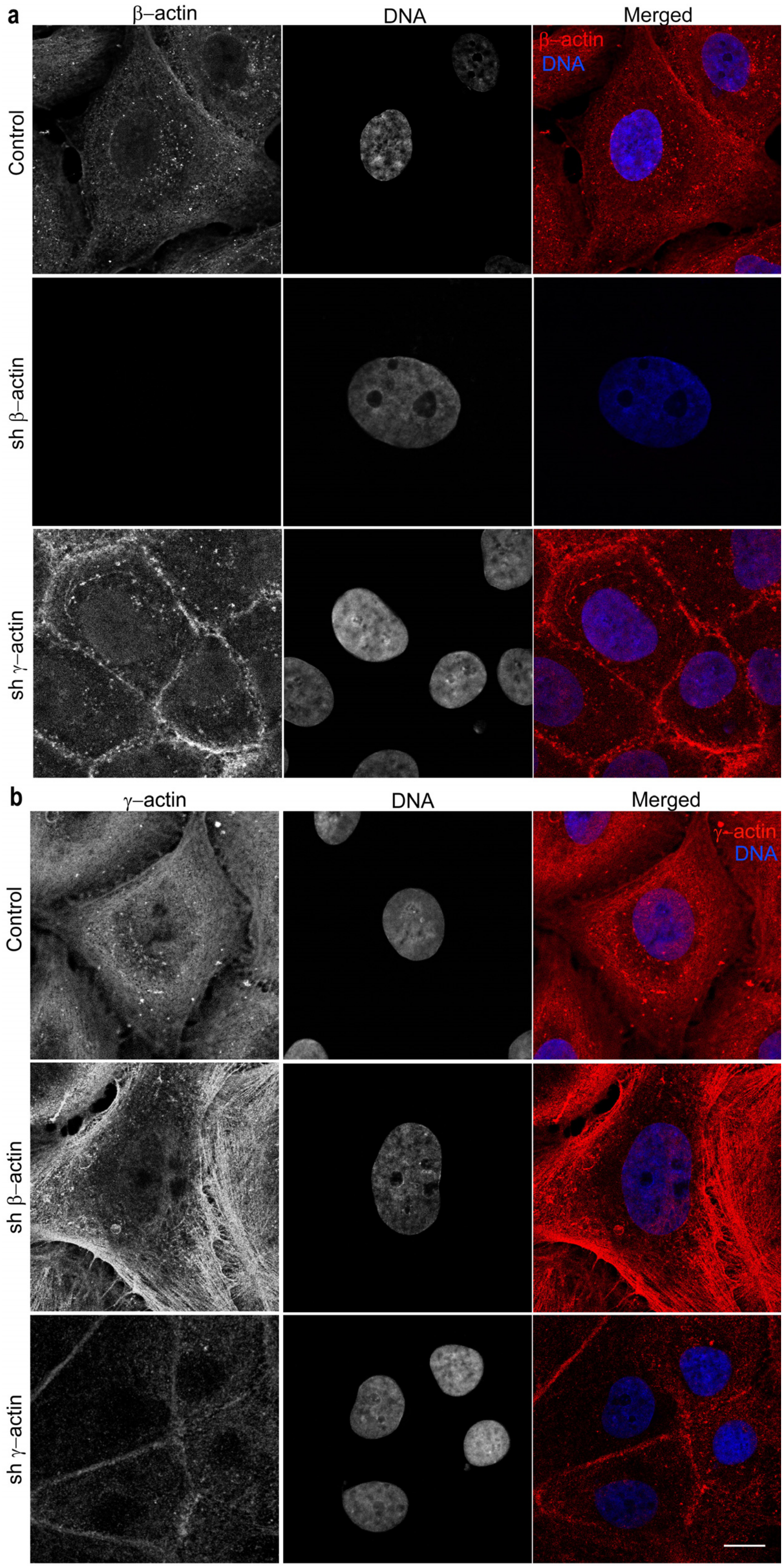

Figure S1. Selective non-muscle actin suppression in A549 cells. (**a**) β-actin; (**b**) γ-actin; laser scanning microscopy, scale bar - 10 μm.

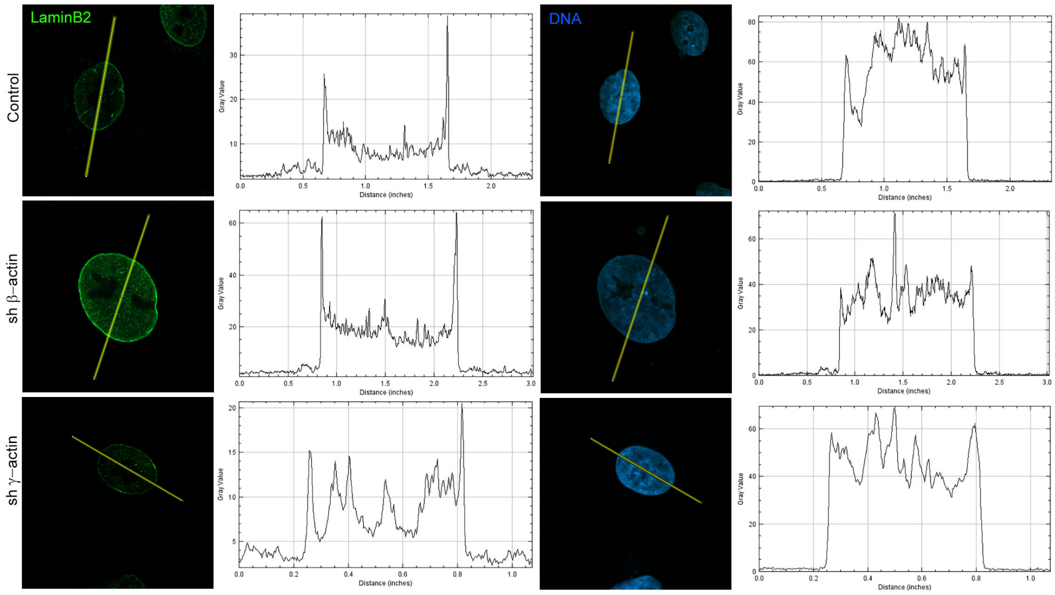

Figure S2. Linescan analysis of lamin B2 in A549 cells following the suppression of non-muscle actin.
